# RNA N1-methyladenosine regulator-mediated methylation modification patterns and heterogeneous signatures in glioma

**DOI:** 10.3389/fimmu.2022.948630

**Published:** 2022-07-22

**Authors:** Meng Mao, Qinjun Chu, Yongli Lou, Peipei Lv, Lin-jian Wang

**Affiliations:** ^1^ Department of Anesthesiology and Perioperative Medicine, Zhengzhou Central Hospital Affiliated to Zhengzhou University, Zhengzhou, China; ^2^ Research of Trauma Center, Zhengzhou Central Hospital Affiliated to Zhengzhou University, Zhengzhou, China; ^3^ Center for Advanced Medicine, College of Medicine, Zhengzhou University, Zhengzhou, China; ^4^ Department of Neurosurgery, Zhengzhou Central Hospital Affiliated to Zhengzhou University, Zhengzhou, China; ^5^ Department of Radiology, Zhengzhou Central Hospital Affiliated to Zhengzhou University, Zhengzhou, China

**Keywords:** glioma, m^1^A, heterogeneity, stemness, tumor microenvironment, TMZ-resistance

## Abstract

N1-methyladenosine (m^1^A) is ubiquitous in eukaryotic RNA and regulates mRNA translation. However, little is known about its regulatory role in glioma. Here, we identified 4 m^1^A modification-related patterns based on m^1^A regulators in the TCGA (The Cancer Genome Atlas) and CGGA (Chinese Glioma Genome Atlas) cohorts. The differences in survival prognosis between different clusters were striking. In addition, stemness, genomic heterogeneity, tumor microenvironment (TME), and immune cell infiltration were also significantly different between the poor and best prognostic clusters. To reveal the underlying mechanism, differentially expressed genes (DEGs) between the poor and best prognostic clusters were identified, and then were integrated for weighted correlation network analysis (WGCNA). After Univariate Cox-LASSO-Multivariate Cox analyses, DEGs PLEK2 and ABCC3 were screened as the risk-hub genes and were selected to construct an m^1^A-related signature. Moreover, ABCC3 exacerbated glioma proliferation and was associated with temozolomide (TMZ) resistance. Overall, our study provided new insights into the function and potential therapeutic role of m^1^A in glioma.

## Introduction

Gliomas are the most common neurological malignancies (1). Currently, surgery followed by chemoradiation is still the standard treatment, but the prognosis is poor (2). In addition, preliminary results from a phase III clinical trial in recurrent glioma suggested that immune checkpoint inhibitor therapy, which has shown promise in tumor therapy, did not significantly improve patient survival (3–6). Extensive intra-tumor and inter-tumor heterogeneity, tumor microenvironment (TME) are particularly critical factors in drug resistance and treatment failure (7, 8).

To date, more than 160 different types of post-transcriptional modifications have been identified on RNA (9). Breakthroughs in sequencing technologies have greatly improved our knowledge of the location, regulation, and function of RNA modifications in the transcriptome (10, 11), which lead to the birth of epitranscriptomics (12). Notably, RNA modifications, especially N6-methyladenosine (m^6^A), 5-methylcytidine (m^5^C) and pseudouridine (Ψ) modification, affect glioma prognosis and have been proposed as a new class of epigenetic markers for the diagnosis of glioma (13–15). Importantly, a recently identified modification, N1-methyladenosine (m^1^A), is ubiquitous in eukaryotic tRNA and rRNA, and recent studies have shown that m^1^A modification can also regulate mRNA translation (16). However, its specific role in glioma remains unclear.

m^1^A modification is dynamically regulated in mammalian RNAs. NML, TRMT6, TRMT10C, TRMT61A, and TRMT61B are identified as methylases (16, 17), besides, ALKBH1 and ALKBH3 are responsible for demethylation (18, 19), and YTHDC1, YTHDF1, YTHDF2 and YTHDF3 are m^1^A binding proteins (20, 21). Here, we identified 4 modification-related clusters based on these m^1^A regulators in the TCGA and CGGA cohorts. Poor- and best-prognostic clusters differed significantly in stemness, genomic heterogeneity, tumor microenvironment (TME) and immune cell infiltration. An m^1^A-related signature was constructed using the risk-hub differentially expressed genes (DEGs) PLEK2 and ABCC3, which were identified between clusters with best and worse prognosis and were highly associated with heterogeneity. Furthermore, we found that ABCC3 was significantly associated with temozolomide (TMZ) resistance, and silencing ABCC3 effectively inhibited glioma cell proliferation. Overall, our study focused on the function role of m^1^A and provided a potential therapeutic strategy for glioma.

## Methods

### Datasets and samples

The TCGA dataset (674 patients included) was downloaded from University of California Santa Cruz (UCSC) Xena browser (https://xenabrowser.net/datapages/) (22), the CGGA #325 (309 patients included) and CGGA #693 (657 patients included) datasets were obtained from the Chinese Glioma Genome Atlas (CGGA) data portal (http://www.cgga.org.cn/) (23), the data for IMvigor210 cohort (348 patients included) were loaded from R package “IMvigor210CoreBiologies” (24), and the GSE148740, GSE113510 and GSE68071 datasets were downloaded from GEO website (https://www.ncbi.nlm.nih.gov/geo/).

### Consensus clustering analysis

Cluster analysis was performed by ConsensusClusterPlus (25), using agglomerative pam clustering with a 1-pearson correlation distances and resampling 80% of the samples for 10 repetitions. The optimal number of clusters was determined using the empirical cumulative distribution function plot.

### Analysis of stemness features

The glioma stemness scores based on RNA expression (RNAss), Epigenetically regulated RNA expression (EREG-EXPss), DNA methylation (DNAss), Epigenetically regulated DNA methylation (EREG-METHs), Differentially methylated probes (DMPss), Enhancer Elements/DNA methylation (ENHss) were calculated according to previous study (26).

### Analysis of genomic heterogeneity

The dataset of glioma simple nucleotide variations processed with MuTect2 software (27) was downloaded from GDC (https://portal.gdc.cancer.gov/). TMB (Tumor mutation burden) and MATH (Mutant-allele tumor heterogeneity) for each glioma were calculated using the tmb function and inferHeterogeneity function of the R package “maftools”, respectively. MSI (Microsatellite instability), Neoantigen, purity, ploidy, HRD (Homologous recombination deficiency) and LOH (Loss of heterozygosity) for each glioma were derived from previous studies (28, 29).

### Immune microenvironment analysis

The stromal, immune, and ESTIMATE scores of each glioma were calculated based on gene expression using the R package “estimate” (30). The abundance of tumor-infiltrating immune cells in glioma were analyzed using the CIBERSOR algorithm on the TIMER2 platform (http://timer.cistrome.org/) (31).

### DEGs screening and enrichment Analysis

Differentially expressed genes (DEGs) between different clusters were screened using the R package” limma” (p< 0.05 and |log_2_FC| ≥ 1) (32). GO analysis of DEGs was performed using Metascape (33). The GSEA (Gene set enrichment analysis) software was downloaded from the GSEA website (http://software.broadinstitute.org/gsea/index.jsp) (34), and the c2.cp.kegg.v7.4.symbols.gmt subset was downloaded from the Molecular Signatures Database (http://www.gsea-msigdb.org/gsea/downloads.jsp) to evaluate related pathways and molecular mechanisms (35).

### Construction of risk signature

Weighted correlation network analysis (WGCNA) was performed to construct the scale-free co-expression network using the R software package “WGCNA”, Six co-expression modules were finally obtained after merging modules with distances less than 0.25. Genes with high connectivity in the significant clinical module were identified as hub genes. Univariate, LASSO, and multivariate regression analysis were then sequentially performed to screen for positive hub genes significantly associated with overall survival (OS). The risk score was calculated as follows:


$$Risk\;score=\sum_{i=1}^{n}\left(Coef_{i}*Exp_{i}\right)$$

### Cell viability analysis

ABCC3 was knocked down using siRNAs (**Supplementary Table 1**, Shanghai GenePharma Co., Ltd) in LN229 and U87 cells. Control and ABCC3-deficient cells were seed into the 96-well plates or glass bottom cell culture dishes. Cell viability was detected using the CCK-8 kit (Dojindo Molecular Technologies) and EdU kit (Beyotime Biotecnology) according to the manufacturer’s instructions.

### Statistical analysis

One-way ANOVA, t test and wilcoxon test were used to analyze the significance of differences in heterogeneity and gene expression. Kaplan-Meier analysis was performed using the “survfit” function of the R package “survival”, and the logrank test method was used to evaluate the significance of the prognostic differences between samples from different groups. ROC analyses for 1-, 3-, 5-year time points were performed using the “roc” function of the R package “pROC”, and the AUC and confidence intervals were evaluated using the “ci” function to obtain the final AUC results. GraphPad Prism and R software were used for all statistical analyses, and p values less than 0.05 were considered statistically significant.

## Results

### Analysis of m^1^A regulators in glioma

**Figure 1:**
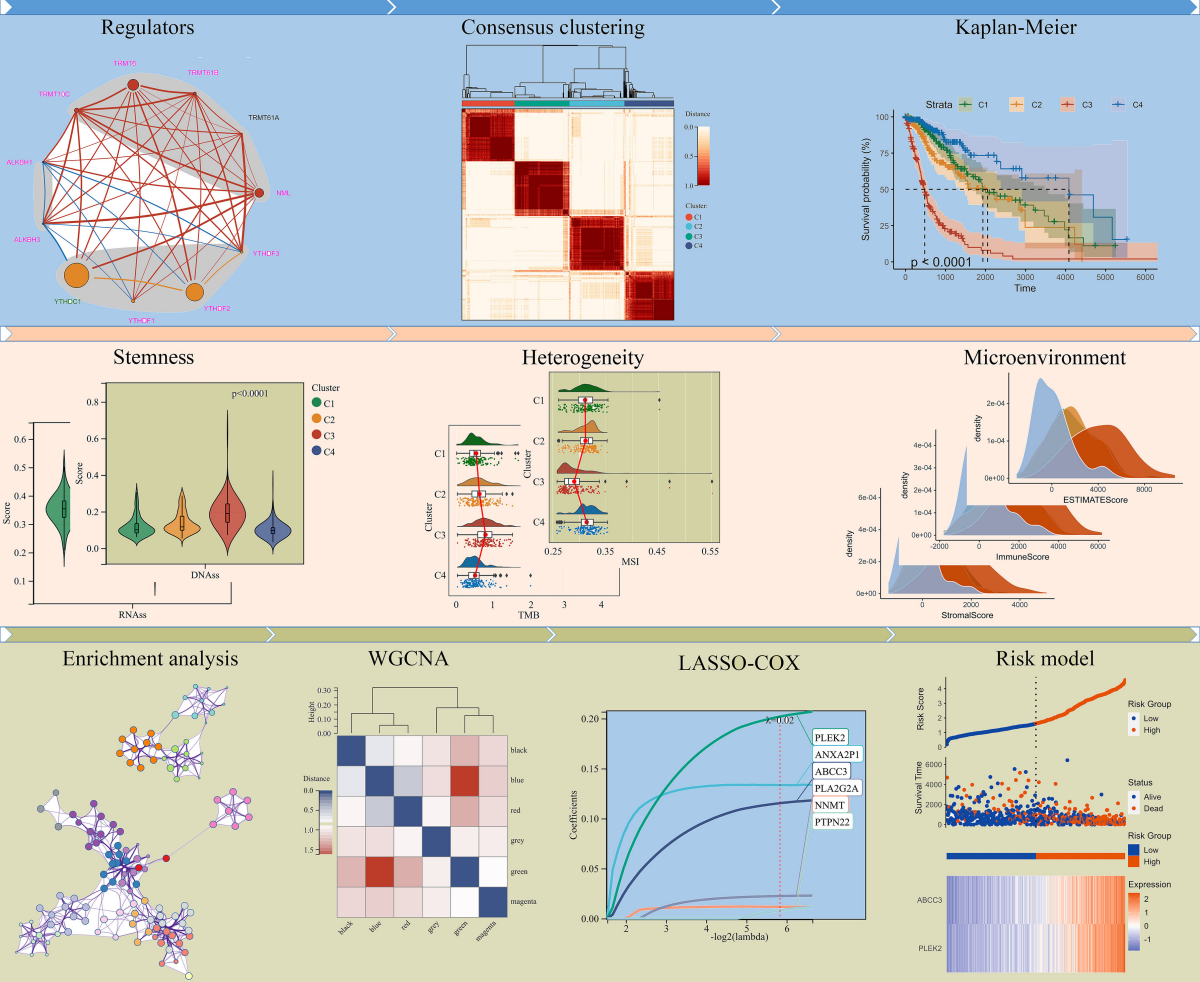
Flowchart of this study.

**Figure 2:**
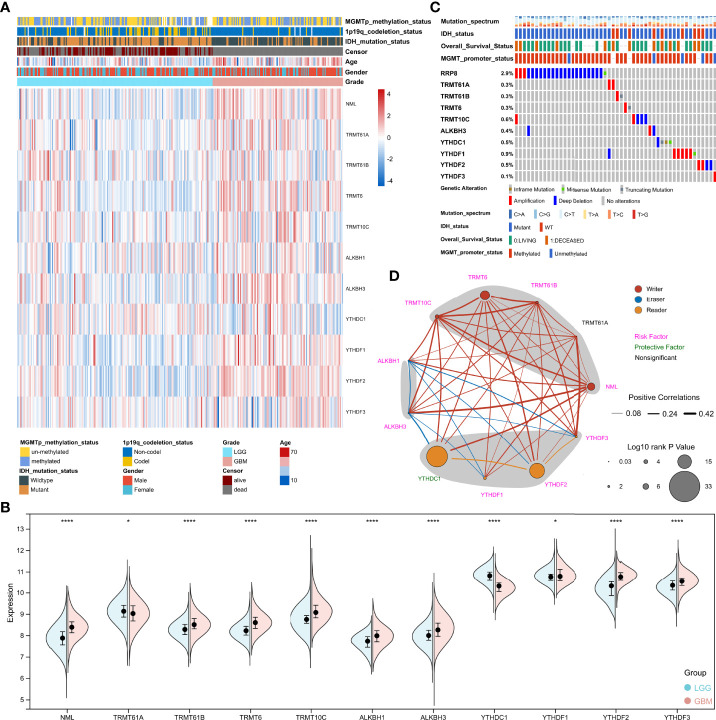
Analysis of m^1^A regulators in glioma. **(A)** Heatmap depicting the expression of m^1^A regulators in the TCGA cohort. **(B)** Spilt violin showing the expression of m^1^A regulators between LGG and GBM. **(C)** Mutation waterfall chart displaying the mutation on m^1^A regulators in glioma. **(D)** The network displaying the relationship and prognosis information of m^1^A regulators. *, P< 0.05; ****, P< 0.0001.

N1-methyladenosine is a unique methylation modification which is ubiquitous and functional in mammalian RNAs. Following the analysis procedure of this study (**Figure 1**), we first analyzed the expression of m^1^A regulators between LGG (Low grade glioma) and GBM (Glioblastoma). The results showed that all regulators were differentially expressed between LGG and GBM in the TCGA cohort (**Figures 2A, B**). Furthermore, except for TRMT61A and ALKBH1, all regulators were also differentially expressed between IDH mutant and IDH wild-type groups (**Supplementary Figure 1**). The mutational landscape of m^1^A regulators in glioma was analyzed and displayed as a waterfall plot, with NML mutations present in 2.9% of the samples (**Figure 2C**). We then studied the interrelationships between m^1^A regulators and their prognostic roles in the TCGA cohort. Results showed that the expression levels of the regulators were significantly related. (**Figure 2D**). In addition to TRMT61A, the expression of other m^1^A regulators was associated with overall survival (OS) in glioma (**Figure 2D**).

### Consensus clustering analysis of m^1^A regulators in glioma

**Figure 3:**
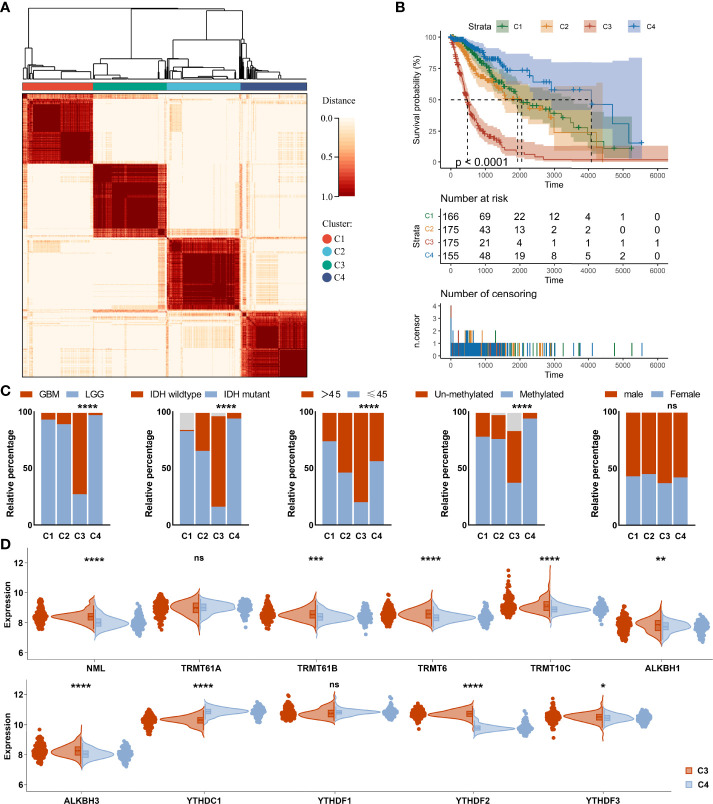
Consensus clustering analysis of m^1^A regulators in glioma. **(A)** Consensus clustering matrix for the most suitable k = 4. **(B)** Kaplan-Meier curves displaying prognostic differences between different clusters. **(C)** Differences in clinical phenotypes between different clusters. **(D)** Differences in expression of m^1^A regulators between cluster 3 and cluster 4. ns, no significance; *, P< 0.05; **, P< 0.01; ***, P< 0.001; ****, P< 0.0001.

Based on m^1^A regulator expression profiles, an unsupervised consensus clustering analysis was performed to identify distinct subtypes in the TCGA dataset. Four clusters were finally determined using the empirical cumulative distribution function plot (**Figure 3A** and **Supplementary Figure 2**). We next analyzed the prognosis of the four clusters and found that there were significant differences in overall survival between these clusters. Patients in cluster 4 survived longer, while patients in cluster 3 survived obviously shorter (**Figure 3B**). It was consistent with the results obtained in unsupervised consensus analysis of the CGGA dataset (**Supplementary Figures 3**, **4**). There were also differences in clinical phenotypes between four clusters, especially the number of GBM, IDH wild-type, older, and MGMT un-methylated phenotypes in cluster 3 (**Figure 3C**). Furthermore, we studied the expression of m^1^A regulators in different clusters. Results displayed that comparing with cluster 4, NML, TRMT61B, TRMT6, TRMT10C, ALKBH1, ALKBH3, YTHDF2, and YTHDF3 were up-regulated, while YTHDC1 was significantly down-regulated in cluster 3 (**Figure 3D**).

### Stemness, genomic heterogeneity and immune microenvironment analysis in different clusters

**Figure 4:**
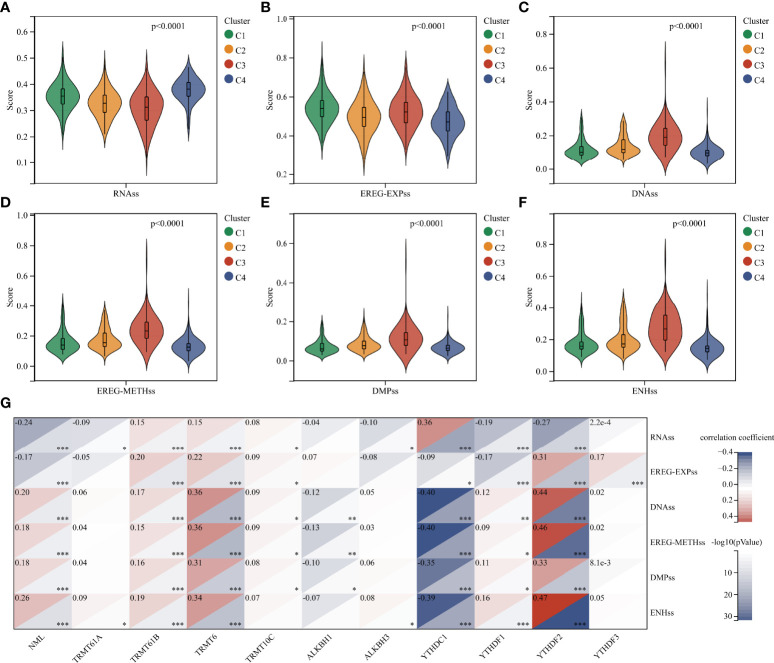
Analysis of stemness features in glioma. **(A–F)** Difference in stemness scores based on RNA expression and DNA methylation between clusters. **(G)** Correlation between m^1^A regulators and stemness scores. *, P < 0.05; **, P < 0.01; ***, P < 0.001.

Tumor progression involves a progressive loss of a differentiated phenotype and the acquisition of progenitor-like, stem-like characteristics. In this study, glioma stemness scores were calculated based on RNA expression and DNA methylation, respectively. The worst-prognostic cluster 3 had lower RNAss but higher EREG-EXPss compared to best-prognostic cluster 4 (**Figure 4A**, **B**). Whereas, all stemness scores according to DNA methylation, such as DNAs, EREG-METHs, DMPss, and ENHss, increased in cluster 3 (**Figure 4C–F**). Correlation analysis showed that m^1^A regulators were highly associated with stemness scores in the TCGA cohort, especially m^1^A writers NML, TRMT61B, TRMT6 and readers YTHDC1, YTHDF1, and YTHDF2 (**Figure 4G**).

**Figure 5:**
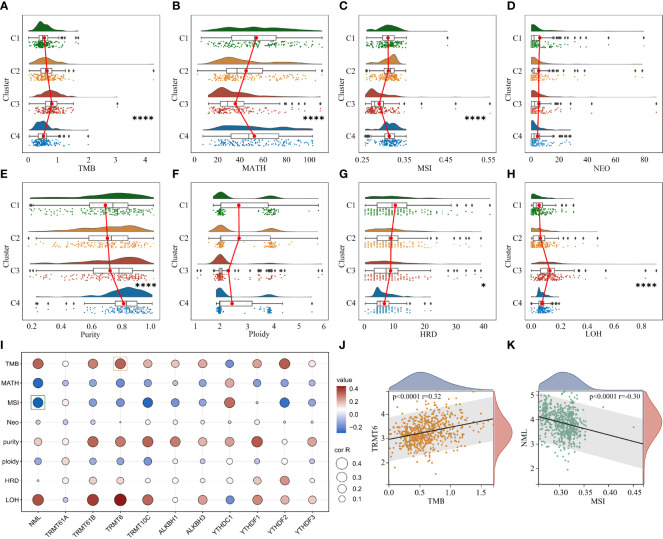
Analysis of genomic heterogeneity in glioma. **(A–H)** Differences in TMB, MATH, MSI, NEO, Purity, Ploidy, HRD and LOH between clusters. **(I)** Correlation between m^1^A regulators and genomic heterogeneity. **(J)** Representative scatter plot showing the correlation between TRMT6 and TMB. **(K)** Representative scatter plot showing the correlation between NML and MSI. *, P < 0.05; ****, P < 0.0001.

Extensive intratumoral and interpatient heterogeneity is an underlying cause of treatment failure, especially for the most aggressive and treatment-resistant GBM (8). Here, we analyzed TMB, MATH, MSI, Neoantigen, purity, ploidy, HRD, and LOH of each glioma in different clusters (**Figure 5**). Cluster 3 with the worst prognosis had higher TMB and LOH than other clusters, while MATH and MSI were lowest (**Figures 5A** **–** **H**). Furthermore, cluster 3 had lower Purity and higher HRD compared to cluster 4 (**Figures 5E**, **G**). Correlation analysis showed that m^1^A regulators were remarkably associated with these heterogenetic scores in the TCGA cohort (**Figure 5I**). For instance, the m^1^A writers TRMT6 and NML were most highly related to TMB and MSI, respectively (**Figures 5J**, **K**).

**Figure 6:**
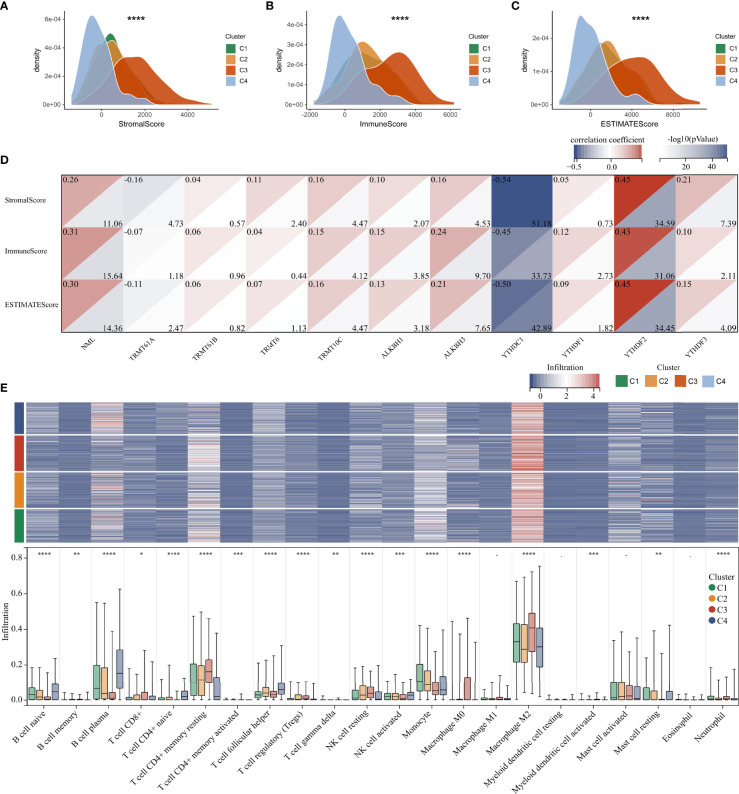
Immune microenvironment analysis in glioma. **(A–C)** Differences in Stromal, Immune, and ESTIMATE scores between clusters. **(D)** Correlation between m^1^A regulators and microenvironment scores. **(E)** Heatmap and boxplot showing the infiltration of 22 immune cells between different clusters. *, P< 0.05; **, P< 0.01; ***, P< 0.001; ****, P< 0.0001.

TME is involved in tumor survival, malignant progression, metastasis and therapy resistance. Therefore, we compared the immune microenvironment of gliomas in different clusters. There were obviously differences in StromalScore, ImmuneScore, and ESTIMATEScore between clusters, with cluster 3 being the highest and cluster 4 being the lowest (**Figures 6A** **–** **C**). Furthermore, m^1^A regulators were tightly associated with StromalScore, ImmuneScore, and ESTIMATEScore, especially the writers NML and TRMT10C, the eraser ALKBH3, and the readers YTHDC1 and YTHDF1 (**Figure 6D**). Analysis of tumor-infiltrating immune cells showed that the abundance of B cell naïve, B cell plasma, Monocyte, and Mast cell resting was dramatically reduced in cluster 3, while Macrophage M0 and Macrophage M2 were dramatically increased (**Figure 6E**).

We re-analyzed stemness, genomic heterogeneity, and immune microenvironment in the LGG subgroup separately. As shown in the **Supplementary Figure 5A**, there was a clear difference in the OS between cluster 3 and cluster 4. Moreover, the stemness score RNAss decreased in the worst-prognostic cluster 3 compared to the best prognostic cluster 4 (**Supplementary Figure 5B**), whereas DNAs, EREG-METHs, DMPss, and ENHss, increased in worst-prognostic cluster 3 (**Supplementary Figures 5C–G**), which were consistent with the entire cohort. Likewise, the genomic heterogeneity analysis (**Supplementary Figures 5H–L**) and immune microenvironment analysis (**Supplementary Figures 5M–O**) yielded similar results to the entire cohort. In addition to the differentially infiltrating immune cells identified between clusters 3 and 4 across the entire cohort, we also found T cell CD4+ naïve, T cell CD4+ memory resting, T cell follicular helper, T cell regulatory (Tregs), Myeloid dendritic cell activated, and Neutrophil were differentially infiltrated between cluster 3 and 4 in the LGG subgroup (**Supplementary Figure 5P**). However, Macrophage M0 and Mast cell resting were not significantly different between clusters 3 and 4 (**Supplementary Figure 5P**).

### Construction of the risk score signature

**Figure 7:**
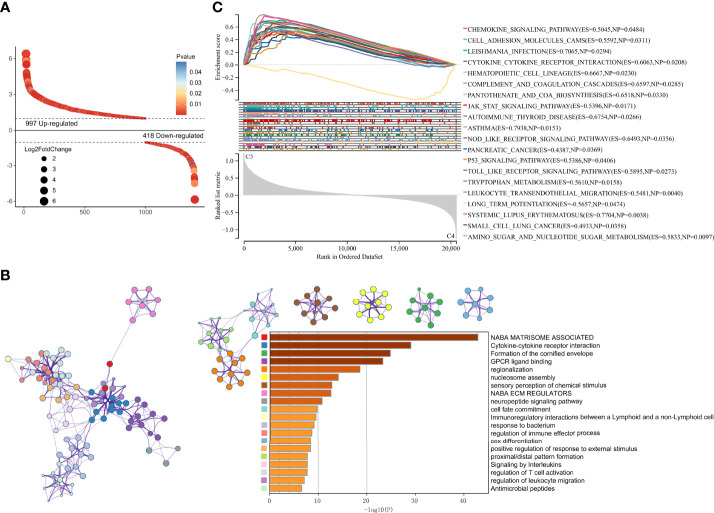
DEGs screening and enrichment Analysis. **(A)** Differentially expressed genes between cluster 3 and cluster 4. **(B)** GO analysis of the differentially expressed genes. **(C)** GSEA analysis of the differentially expressed genes.

To reveal the mechanism leading to differences in prognosis and heterogeneity, DEGs between cluster 3 and cluster 4 were screened using R package “limma”. 997 up-regulated and 418 down-regulated genes were identified (**Figure 7A**). GO enrichment analysis showed that the DEGs were enriched in the Cytokine-cytokine receptor interaction and Immunoregulatory interactions (**Figure 7B**). GSEA analysis showed the DEGs were enriched in CELL ADHESION MOLECULES CAMS, JAK STAT signaling pathway, P53 signaling pathway, and immune-related pathways (**Figure 7C**).

**Figure 8:**
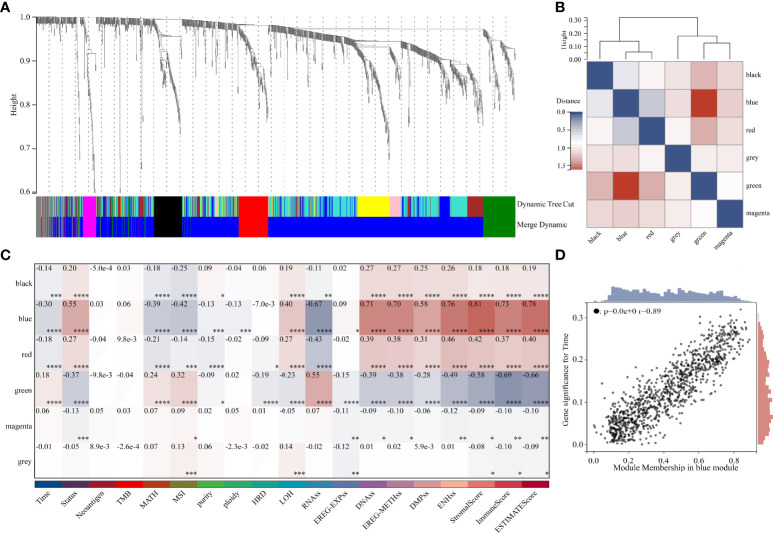
Weighted Correlation Network Analysis. **(A)** Clustering of module genes in the TCGA cohort. **(B)** Cluster dendrogram of modules. **(C)** Module-trait relationships. **(D)** Scatter plot of correlation between GS and MM. *, P < 0.05; **, P < 0.01; ***, P < 0.001; ****, P < 0.0001.

**Figure 9:**
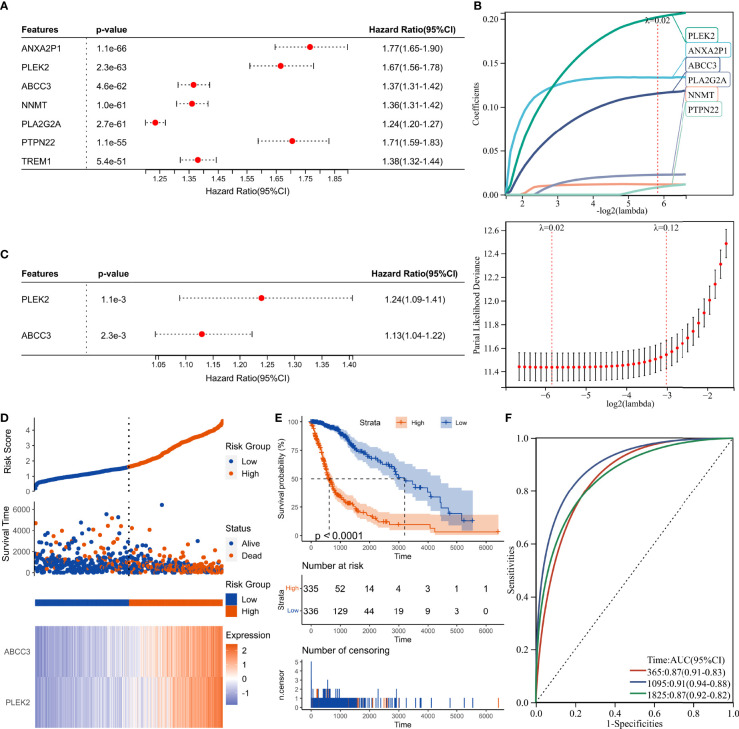
Construction of risk score signature. **(A)** Univariate Cox regression analysis of hub genes in the TCGA cohort. **(B)** LASSO Cox regression analysis of hub genes in the TCGA cohort. **(C)** Multivariate Cox regression analysis of hub genes in the TCGA cohort. **(D)** Distribution of the risk score, survival status, and expression profile of the prognostic genes in the TCGA cohort. **(E)** Kaplan-Meier curves displaying prognostic differences between high- and low-risk groups in the TCGA cohort. **(F)** The ROC curves describing the sensitivity and specificity of risk score in predicting OS at 1-, 3- and 5-year time points in the TCGA cohort.

DEGs and traits such as stemness, genomic heterogeneity, TME were integrated for WGCNA analysis. Six co-expression modules were identified, of which the blue module was most associated with the traits (**Figure 8**). Seven genes with a module membership greater than 0.85 were identified as hub genes in the blue module. We then sequentially performed Univariate, LASSO, and Multivariate Cox regression analysis to filter variables and reduce model complexity (**Figures 9A–C**). PLEK2 and ABCC3 were ultimately selected as risk-hub genes (**Figure 9C**) and used to construct a risk model (**Figure 9D**). Kaplan Meier curves displayed that higher risk scores were associated with poorer prognosis (**Figure 9E**). Moreover, the sensitivity and specificity of risk score was greatly high in predicting the survival of glioma patients at 1-, 3- and 5-years (**Figure 9F**). The results of the risk model were also validated in the CGGA #325 and CGGA #693 datasets (**Supplementary Figure 6**), as well as the LGG subgroup (**Supplementary Figure 7**).

### ABCC3 is associated with tumor stemness, genomic heterogeneity, immune microenvironment and TMZ-resistance

**Figure 10:**
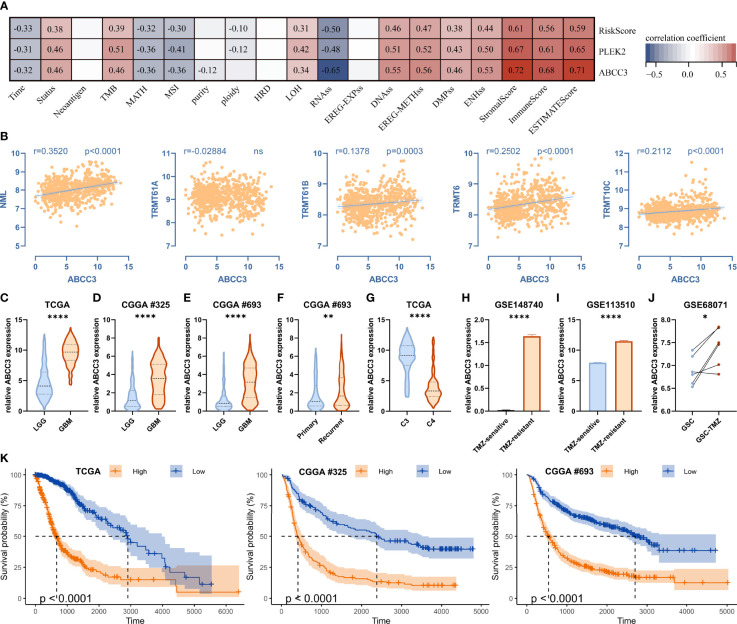
ABCC3 is associated with tumor stemness, genomic heterogeneity, immune microenvironment and TMZ-resistance. **(A)** Correlations between ABCC3 and tumor stemness, genomic heterogeneity, and immune microenvironment. **(B)** Correlation between ABCC3 and m^1^A regulators. **(C–E)** Differences in ABCC3 expression between LGG and GBM in TCGA, CGGA #325 and CGGA #693 cohort, respectively. **(F)** Differences in ABCC3 expression between primary and recurrent gliomas. **(G)** Differences in ABCC3 expression between cluster 3 and cluster 4 in the TCGA cohort. **(H)** Differences in ABCC3 expression between TMZ-sensitive and TMZ-resistant PDX models, **(I)** LN229 cells, and **(J)** GSC cells. **(K)** Kaplan-Meier curves displaying prognostic differences between ABCC3 high and low expression groups in the TCGA, CGGA #325, and CGGA #693 cohort, respectively. *, P < 0.05; **, P < 0.01; ****, P < 0.0001.

We then analyzed the relationship between the traits and the risk score, the prognostic hub genes PLEK2 and ABCC3 (**Figure 10A**). High associations with stemness, genomic heterogeneity and TME suggested that risk scores might be associated with immunotherapy outcome. Therefore, we selected the most widely used IMVigor210 ICBs cohort, which contains detailed clinicopathological profiles and gene expression data of a large number of patients (n=348) receiving immune checkpoint blockade therapy, to validate the m^1^A signature. Results showed that the low-risk group had more responders than the high-risk group, and the risk scores of the CR/PR group were lower than that of the SD/PD group (**Supplementary Figure 8**). Moreover, low-risk patients had better overall survival (**Supplementary Figure 8**).

In addition to being associated with stemness, genomic heterogeneity, and TME (**Figure 10A**), ABCC3 was also significantly correlated with the expression of m^1^A writers such as NML, TRMT61B, TRMT6 and TRMT10C (**Figure 10B**). Additionally, ABCC3 was up-regulated in the poor-prognostic cluster 3 relative to cluster 4 (**Figures 7A** and **10G**), and it was also up-regulated in GBM relative to LGG in the TCGA, CGGA #325 and CGGA #693 cohorts (**Figures 10C–E**). ABCC3 is a member of the MRP subfamily which is involved in multi-drug resistance. We found ABCC3 was up-regulated in the TMZ-resistant PDX model (**Figure 10H**), TMZ-resistant LN229 cells (**Figure 10I**), and TMZ-resistant glioma stem cells (GSCs) (**Figure 10J**) compared to corresponding controls. Furthermore, ABCC3 significantly affected the prognosis of gliomas in the TCGA, CGGA #325, and CGGA #693 cohorts (**Figure 10K**). Therefore, ABCC3 may be involved in the resistance of TMZ.

### ABCC3 affects the proliferation of glioma cells

**Figure 11:**
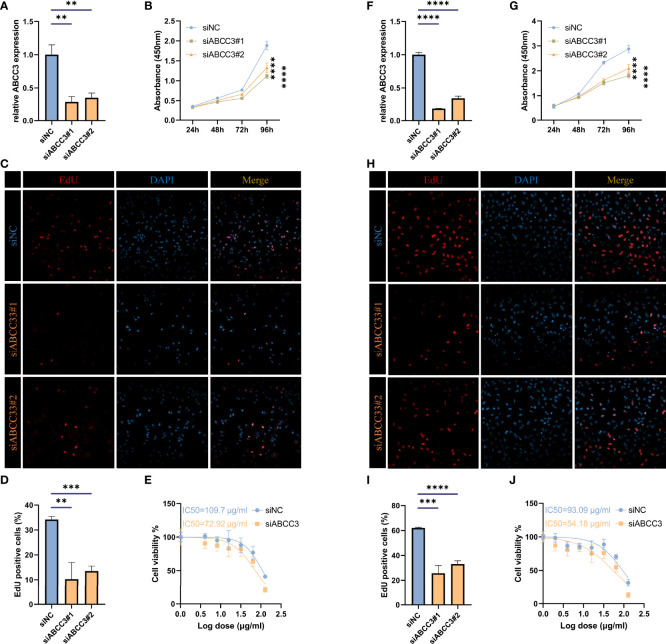
ABCC3 affects the proliferation of glioma cells. **(A)** TIMP1 knockdown efficiency in U87 cells. **(B)** Growth curves of ABCC3-defficient and control U87 cells. **(C, D)** Proportion of EdU positive cells in ABCC3-defficient and control U87 cells. **(E)** IC50 analysis of resistance to TMZ in ABCC3-defficient and control U87 cells. **(F)** TIMP1 knockdown efficiency in LN229 cells. **(G)** Growth curves of ABCC3-defficient and control LN229 cells. **(H–I)** Proportion of EdU positive cells in ABCC3-defficient and control LN229 cells. **(J)** IC50 analysis of resistance to TMZ in ABCC3-defficient and control LN229 cells. **, P < 0.01; ***, P < 0.001; ****, P < 0.0001.

To investigate the effect of ABCC3 on glioma cells, we knocked down it in LN299 and U87 cells, respectively (**Figures 11A, F**). Then we measured the growth curves of the ABCC3-defficient and control cells using the CCK-8 kit (**Figures 11B, G**), and detected the proportion of proliferating cells using the EdU kit (**Figures 11C, D, H, I**). All the results indicated that ABCC3 significantly affected the proliferation of glioma cells. Furthermore, IC50 analysis showed that knockdown of ABCC3 considerably reduced the resistance of glioma cells to TMZ (**Figures 11E, J**).

## Discussion

Epigenetic modifications, such as DNA methylation, histone modifications, can result in heritable phenotypic changes but without any changes in nucleic acid sequence. RNA modifications play a critical role in regulating multiple metabolic processes of RNA, such as localization, transport, splicing, stabilization, and translation, which also influence phenotype, and thus have been proposed as a new class of epigenetic regulators recently (36, 37). Among all mRNA modifications, N6-methyladenosine (m^6^A), 5-methylcytidine (m^5^C) and pseudouridine (Ψ) modification, have been found to be epigenetic markers for the diagnosis of glioma due to their impact on prognosis (13–15). Surprisingly, m^1^A also has such potential, as regulators of m^1^A modification markedly affected glioma prognosis, with NML, TRMT6, TRMT10C, TRMT61B, ALKBH1, ALKBH3, YTHDF1, YTHDF2, and YTHDF3 as risk factors but YTHDC1 as a protective factor (**Figure 2**).

According to consensus clustering analysis of all m^1^A regulators, gliomas could be divided into four clusters with distinct modification patterns (**Figure 3** and **Supplementary Figure 3**). The overall survival of the four clusters was significantly different (**Figure 3B**), suggesting that m^1^A modification patterns might be used to predict prognosis. Molecular characteristics such as IDH mutation status and MGMT methylation status have been used for pathological diagnosis of glioma. In this study, we found these characteristics differed among the four clusters, with the majority of the worst-prognostic group being IDH wildtype and MGMT un-methylated patients (**Figure 3C**). Interestingly, tumor cell stemness, heterogeneity, and microenvironment which are associated with malignant progression and therapy resistance (8) also differed significantly between the best and worst prognostic clusters (**Figures 4** **-** **6**). These results raised some points worth investigating, what’s the relationship between m^1^A modification and prognostic and heterogeneous features; can m^1^A shape tumor heterogeneity? Correlation analyses between regulators and features confirmed the possibility of such a relationship (**Figures 4G**, **5I** and **6D**), but further elaboration is needed.

Herein, PLEK2 and ABCC3 were screened as risk-hub genes for their high connectivity in the significant clinical module as well as their incomparably prognostic roles (**Figures 7**–**9**). Compared with other epigenetic modification risk models, such as m^6^A and m^5^C (15, 38), the risk model constructed in study was very simple but showed high efficiency in TCGA, CGGA #325, and CGGA #693 cohorts (**Figure 9** and **Supplementary Figure 6**). Furthermore, risk model could predict susceptibility to TMZ and outcome of ICBs treatment (**Figures 10**, **11** and **Supplementary Figure 8**).

ABCC3 protein is a member of the ATP-binding cassette transporter that binds and hydrolyzes ATP to enable active transport of various substrates including many toxicants and endogenous compound across extra- and intra-cellular membranes (39–41). ABCC3 protein belongs to the Multidrug Resistance-Associated Protein (MRP) subfamily and confers resistance to various anticancer drugs, methotrexate, tenoposide and etoposide by decreasing accumulation of these drugs in cells (39, 41). ABCC3 was remarkably upregulated in the TMZ-resistant glioma cells (**Figures 10H–J**) and promoted glioma proliferation (**Figure 11**), indicating it might be associated with the poor outcome of GBM receiving standard-of-care for concurrent radiotherapy and TMZ-chemotherapy after surgical resection (2, 42). Tumor heterogeneity, especially immunosuppressive TME, was involved in resistance to immune checkpoint blockers therapy (7). Notably, ABCC3 was closely linked to glioma heterogeneity (**Figure 10A**), low-risk group had more responders than the high-risk group, and low-risk patients had better overall survival (**Supplementary Figure 8**). Therefore, the relationship between ABCC3 expression and the failure of ICBs therapy needs to be studied.

## Conclusions

In conclusion, we comprehensively analyzed the effect of m^1^A on glioma, and provided potential targets for improving standard therapy and immunotherapy in glioma.

## Data availability statement

The datasets presented in this study can be found in online repositories. The names of the repository/repositories and accession number(s) can be found in the article/**supplementary material**.

## Author contributions

MM, QC and L-JW conceived and designed the experiments. YL and PL contributed data analysis. L-JW wrote the manuscript. MM, QC and L-JW approved final version of manuscript. All authors contributed to the article and approved the submitted version.

## Funding

This work was supported by the National Natural Science Foundation of China (82101401).

## Conflict of interest

The authors declare that the research was conducted in the absence of any commercial or financial relationships that could be construed as a potential conflict of interest.

## Publisher’s note

All claims expressed in this article are solely those of the authors and do not necessarily represent those of their affiliated organizations, or those of the publisher, the editors and the reviewers. Any product that may be evaluated in this article, or claim that may be made by its manufacturer, is not guaranteed or endorsed by the publisher.

## Supplementary material

Supplementary Figure 1 Expression of m^1^A regulators in glioma. **(A)** Violin plot showing the expression of m^1^A regulators between IDH mutant and IDH wildtype groups in the TCGA dataset. **(B)** Violin plot showing the expression of m^1^A regulators between LGG and GBM in the CGGA #325 cohort. *, P< 0.05; **, P < 0.01; ***, P < 0.001; ****, P < 0.0001.

Supplementary Figure 2 Consensus clustering analysis of m^1^A regulators in the TCGA dataset. **(A)** Consensus clustering cumulative distribution function for k = 2 to 10. **(B)** Area under the distribution curve for k = 2 to 10. **(C)** Consistency of sample clustering for k = 2 to 10.

Supplementary Figure 3 Consensus clustering analysis of m^1^A regulators in the CGGA #325 dataset. **(A)** Consensus clustering cumulative distribution function for k = 2 to 10. **(B)** Area under the distribution curve for k = 2 to 10. **(C)** Consistency of sample clustering for k = 2 to 10. **(D)** Consensus clustering matrix for the most suitable k = 4.

Supplementary Figure 4 Kaplan-Meier curves displaying prognostic differences between different clusters in the CGGA #325 cohort.

Supplementary Figure 5 Analysis of stemness, genomic heterogeneity and Immune microenvironment features in LGG subgroup. **(A)** Kaplan-Meier curves displaying prognostic differences between cluster 3 and cluster 4 in the LGG subgroup. **(B–G)** Differences in stemness score between cluster 3 and cluster 4 in the LGG subgroup. **(H–L)** Differences in genomic heterogeneity between cluster 3 and cluster 4 in the LGG subgroup. **(M–O)** Differences in immune microenvironment between cluster 3 and cluster 4 in the LGG subgroup. **(P)** Differences in infiltration of 22 immune cells between cluster 3 and cluster 4 in the LGG subgroup. *, P < 0.05; **, P < 0.01; ***, P 0.001; ****, P < 0.0001.

Supplementary Figure 6 Validation of the risk signature in the CGGA #325 and CGGA #693 cohorts. **(A)** Distribution of the risk score, survival status, and expression profile of the prognostic genes in the CGGA #325 cohort. **(B)** Kaplan-Meier curves displaying prognostic differences between high- and low-risk groups in the CGGA #325 cohort. **(C)** The ROC curves describing the sensitivity and specificity of risk score in predicting OS at 1-, 3- and 5-year time points in the CGGA #325 cohort. **(D)** Distribution of the risk score, survival status, and expression profile of the prognostic genes in the CGGA #693 cohort. **(E)** Kaplan-Meier curves displaying prognostic differences between high- and low-risk groups in the CGGA #693 cohort. **(F)** The ROC curves describing the sensitivity and specificity of risk score in predicting OS at 1-, 3- and 5-year time points in the CGGA #693 cohort.

Supplementary Figure 7 Validation of the risk score in LGG subgroup. **(A)** Kaplan-Meier curves displaying prognostic differences between high- and low-risk groups in the LGG subgroup. **(B)** The ROC curves describing the sensitivity and specificity of risk score in predicting OS at 1-, 3- and 5-year time points in the LGG subgroup.

Supplementary Figure 8 Validation of the risk score in the IMvigor210 cohort. **(A)** Boxplot depicting the risk scores between SD/PD and CR/PR groups in the IMvigor210 cohort. **(B)** Differences in the number responders between high- and low-risk groups in the IMvigor210 cohort. **(C)** Kaplan-Meier curves displaying prognostic differences between high- and low-risk groups in the IMvigor210 cohort. *, P < 0.05; **, P < 0.01; ***, P < 0.001; ****, P < 0.0001.

Supplementary Table 1 Primers and siRNAs used in this study.
