# Supplementary figures and images for: RNA N1-methyladenosine regulator-mediated methylation modification patterns and heterogeneous signatures in glioma
Source: Front Immunol. 2022 Jul 22;13:948630. doi: 10.3389/fimmu.2022.948630 (PMC9354098; doi:10.3389/fimmu.2022.948630)

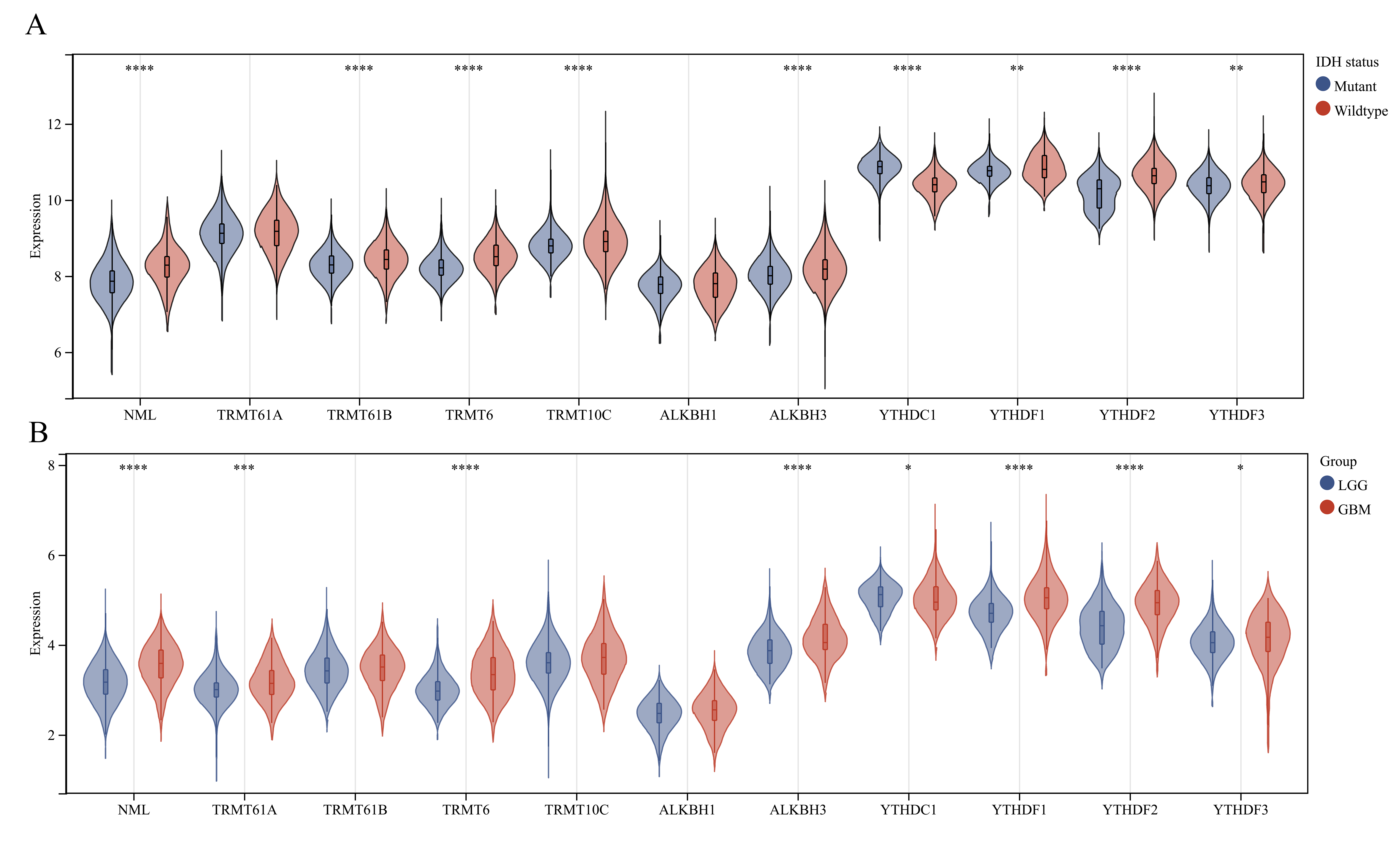

Supplement: Supplementary Figure 1 — Expression of m1A regulators in glioma. (A) Violin plot showing the expression of m1A regulators between IDH mutant and IDH wildtype groups in the TCGA dataset. (B) Violin plot showing the expression of m1A regulators between LGG and GBM in the CGGA #325 cohort. *, P< 0.05; **, P < 0.01; ***, P < 0.001; ****, P < 0.0001. [file Image_1.tif]

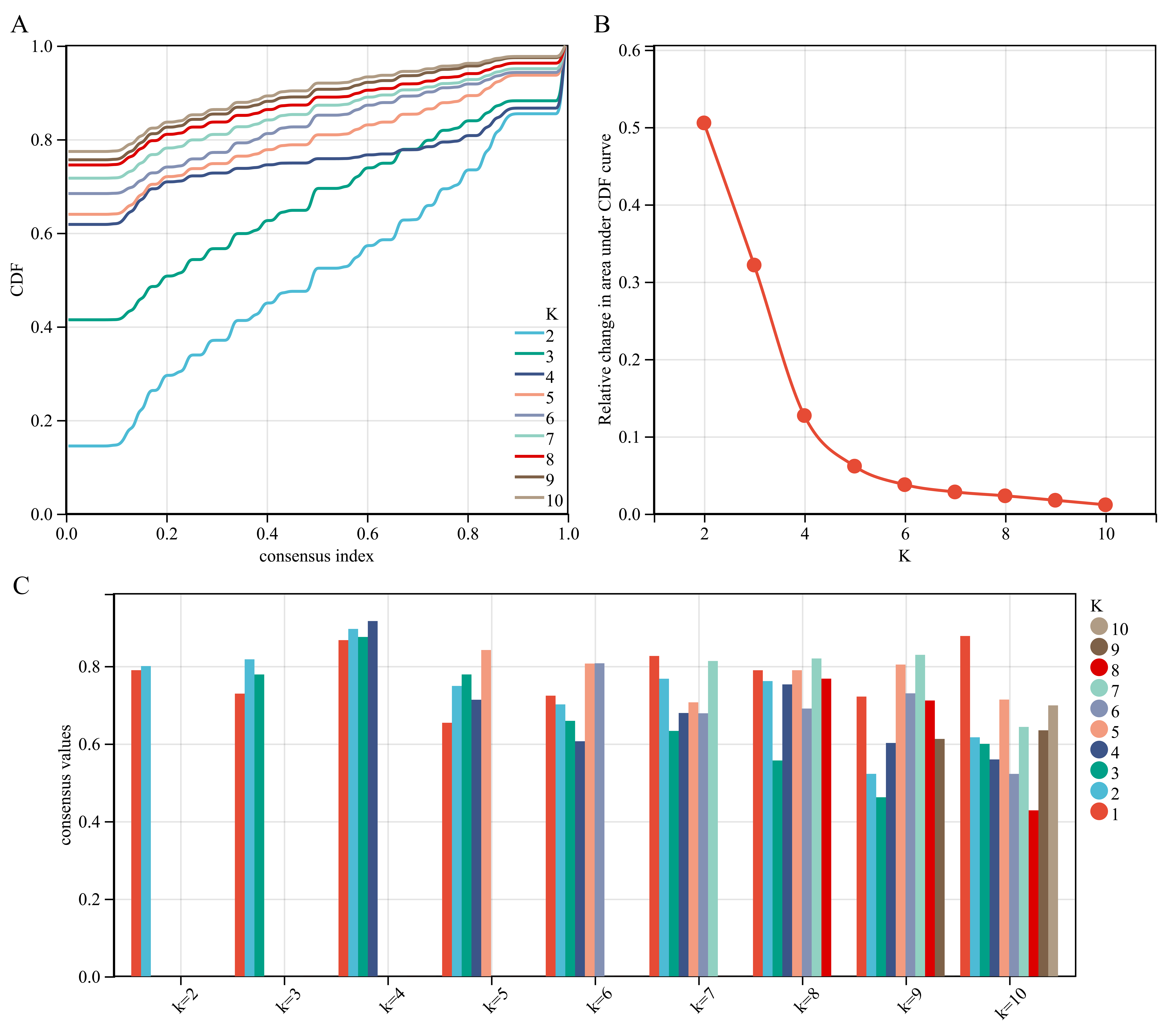

Supplement: Supplementary Figure 2 — Consensus clustering analysis of m1A regulators in the TCGA dataset. (A) Consensus clustering cumulative distribution function for k = 2 to 10. (B) Area under the distribution curve for k = 2 to 10. (C) Consistency of sample clustering for k = 2 to 10. [file Image_2.tif]

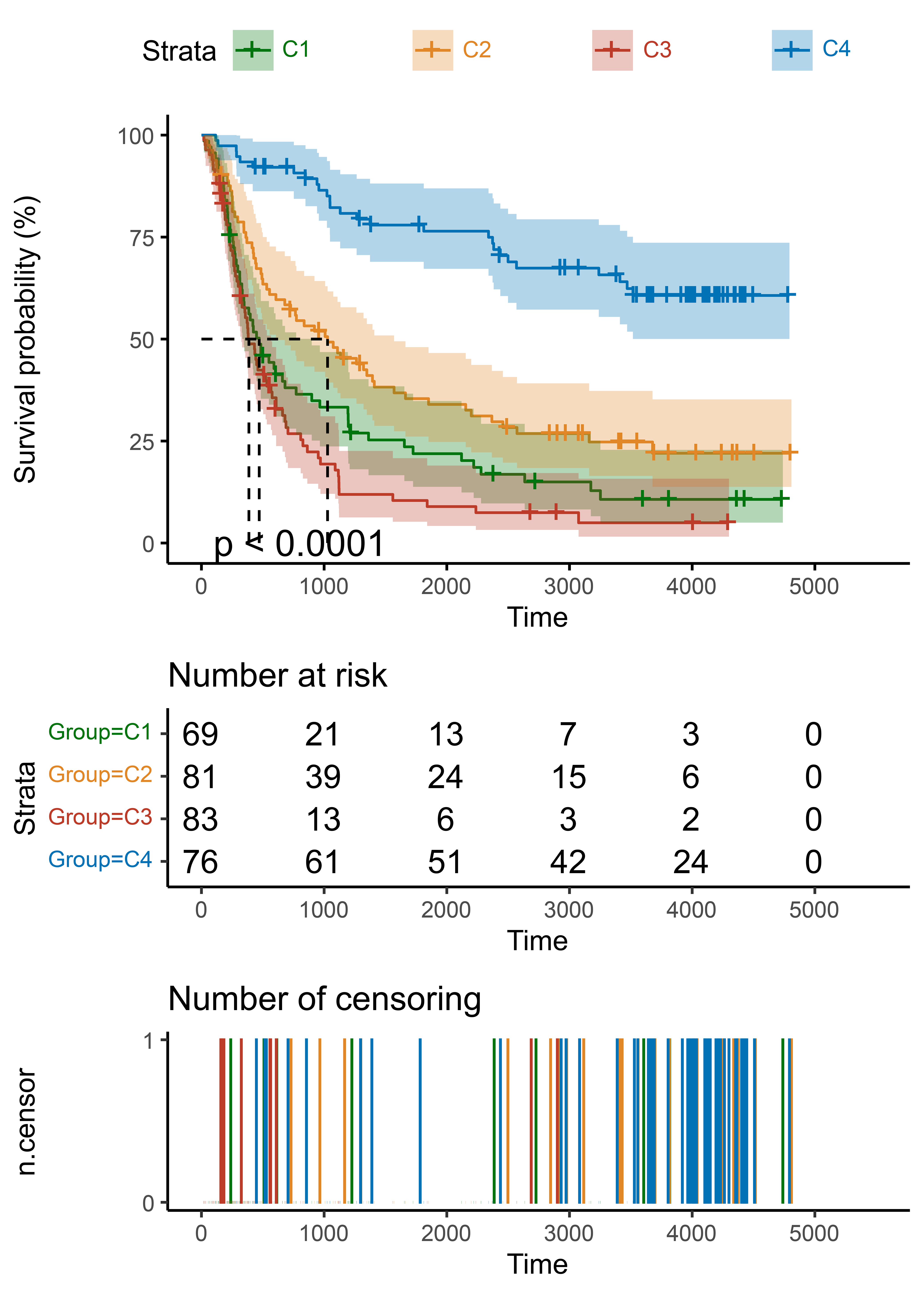

Supplement: Supplementary Figure 4 — Kaplan-Meier curves displaying prognostic differences between different clusters in the CGGA #325 cohort. [file Image_4.tif]

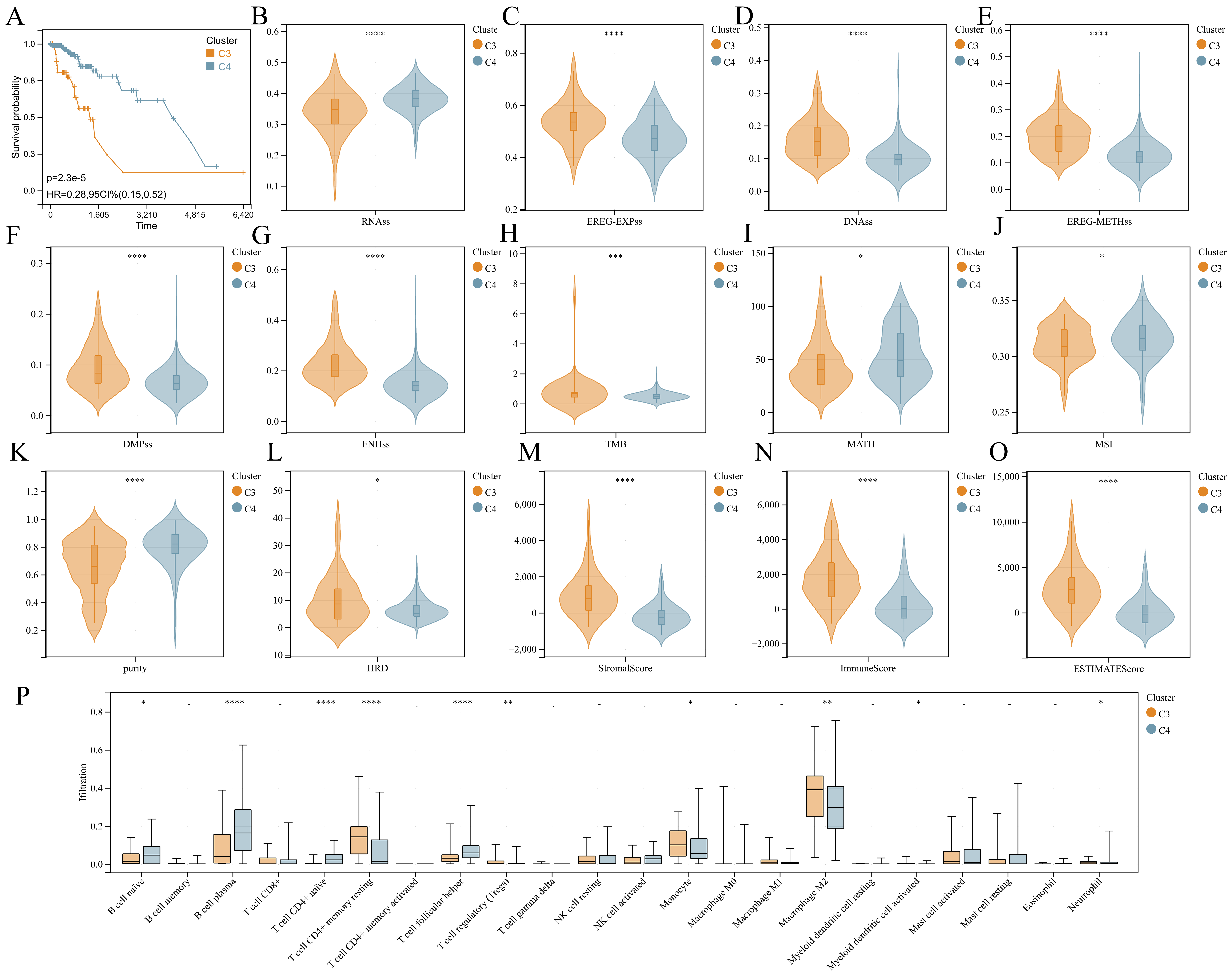

Supplement: Supplementary Figure 5 — Analysis of stemness, genomic heterogeneity and Immune microenvironment features in LGG subgroup. (A) Kaplan-Meier curves displaying prognostic differences between cluster 3 and cluster 4 in the LGG subgroup. (B–G) Differences in stemness score between cluster 3 and cluster 4 in the LGG subgroup. (H–L) Differences in genomic heterogeneity between cluster 3 and cluster 4 in the LGG subgroup. (M–O) Differences in immune microenvironment between cluster 3 and cluster 4 in the LGG subgroup. (P) Differences in infiltration of 22 immune cells between cluster 3 and cluster 4 in the LGG subgroup. *, P < 0.05; **, P < 0.01; ***, P 0.001; ****, P < 0.0001. [file Image_5.tif]

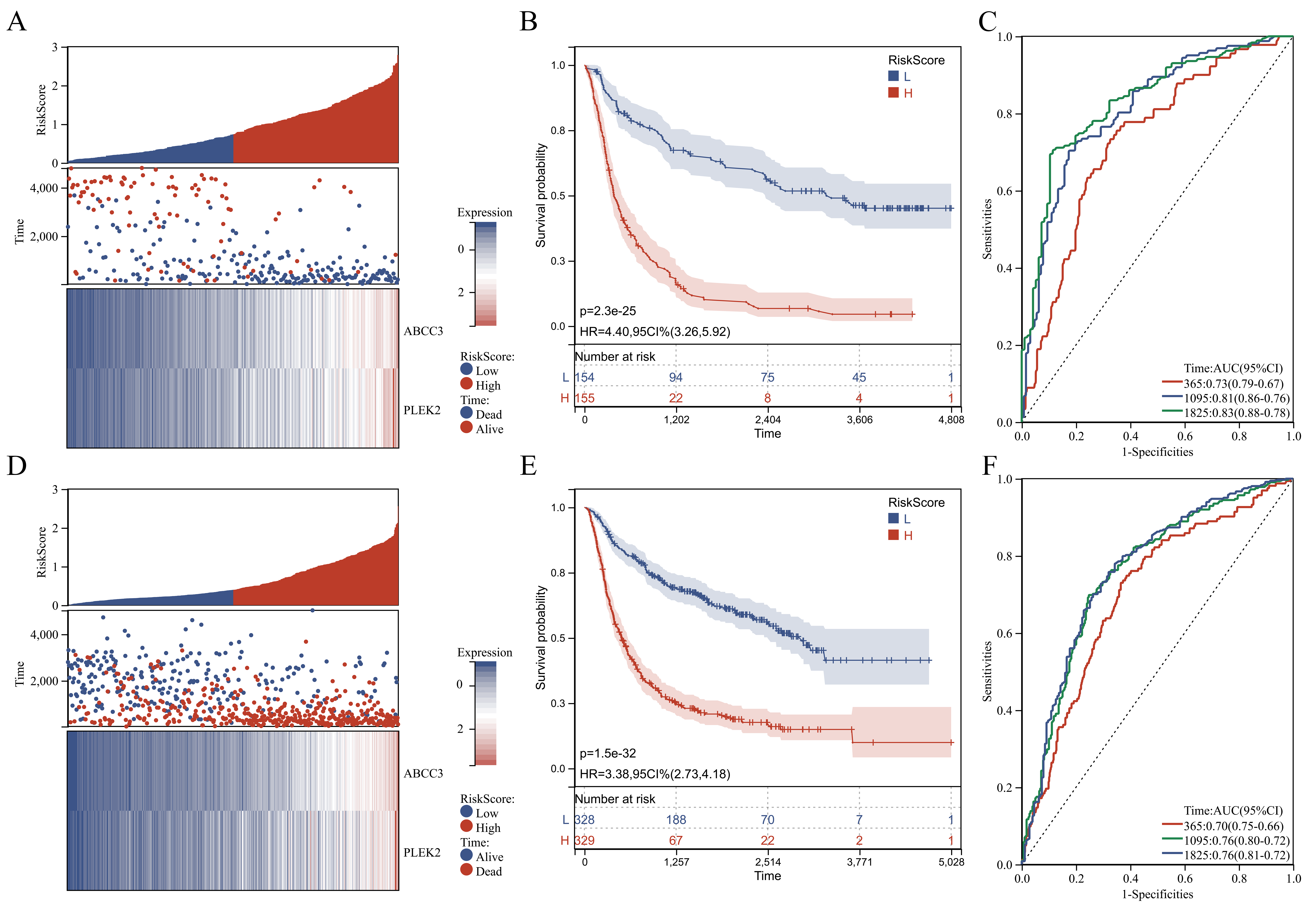

Supplement: Supplementary Figure 6 — Validation of the risk signature in the CGGA #325 and CGGA #693 cohorts. (A) Distribution of the risk score, survival status, and expression profile of the prognostic genes in the CGGA #325 cohort. (B) Kaplan-Meier curves displaying prognostic differences between high- and low-risk groups in the CGGA #325 cohort. (C) The ROC curves describing the sensitivity and specificity of risk score in predicting OS at 1-, 3- and 5-year time points in the CGGA #325 cohort. (D) Distribution of the risk score, survival status, and expression profile of the prognostic genes in the CGGA #693 cohort. (E) Kaplan-Meier curves displaying prognostic differences between high- and low-risk groups in the CGGA #693 cohort. (F) The ROC curves describing the sensitivity and specificity of risk score in predicting OS at 1-, 3- and 5-year time points in the CGGA #693 cohort. [file Image_6.tif]

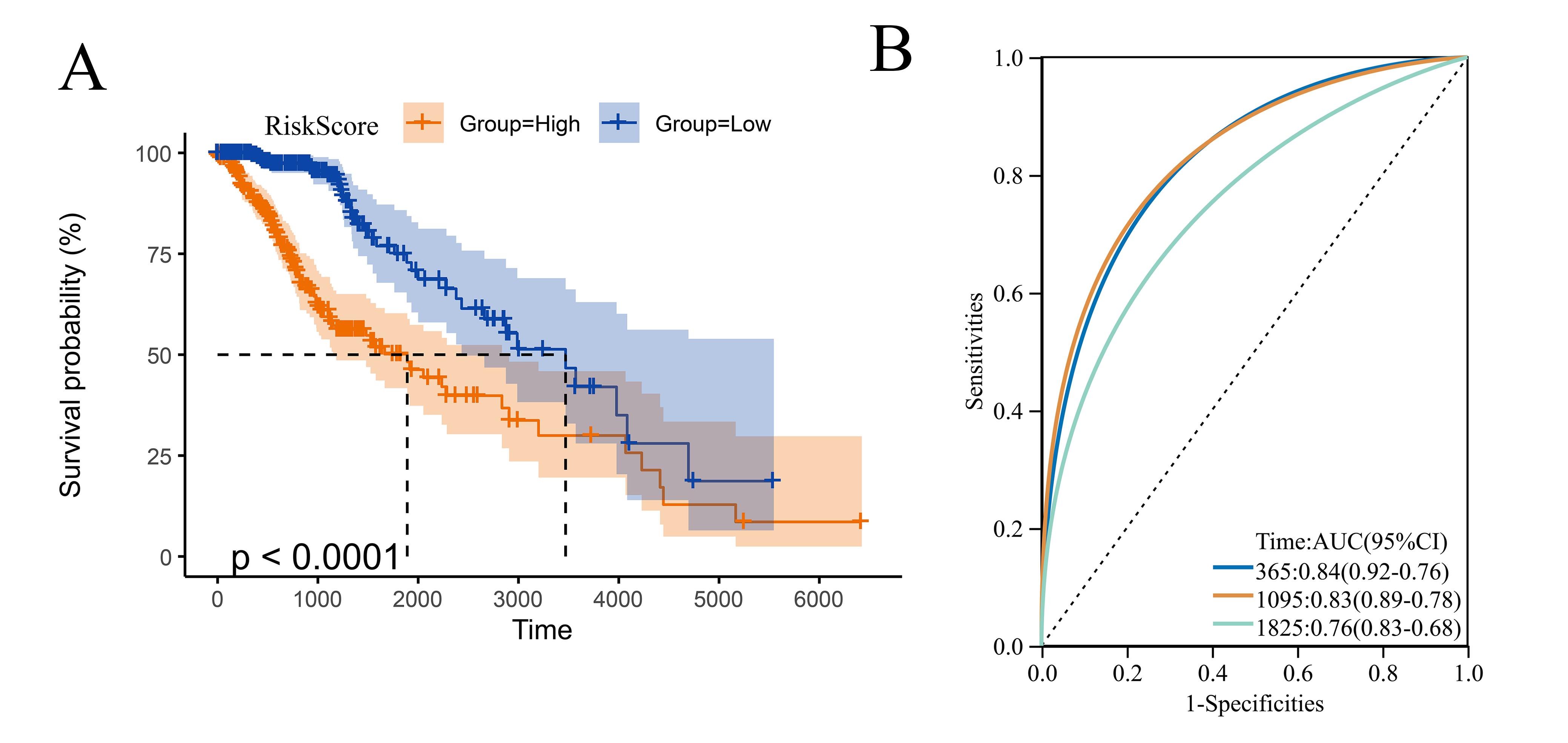

Supplement: Supplementary Figure 7 — Validation of the risk score in LGG subgroup. (A) Kaplan-Meier curves displaying prognostic differences between high- and low-risk groups in the LGG subgroup. (B) The ROC curves describing the sensitivity and specificity of risk score in predicting OS at 1-, 3- and 5-year time points in the LGG subgroup. [file Image_7.tif]

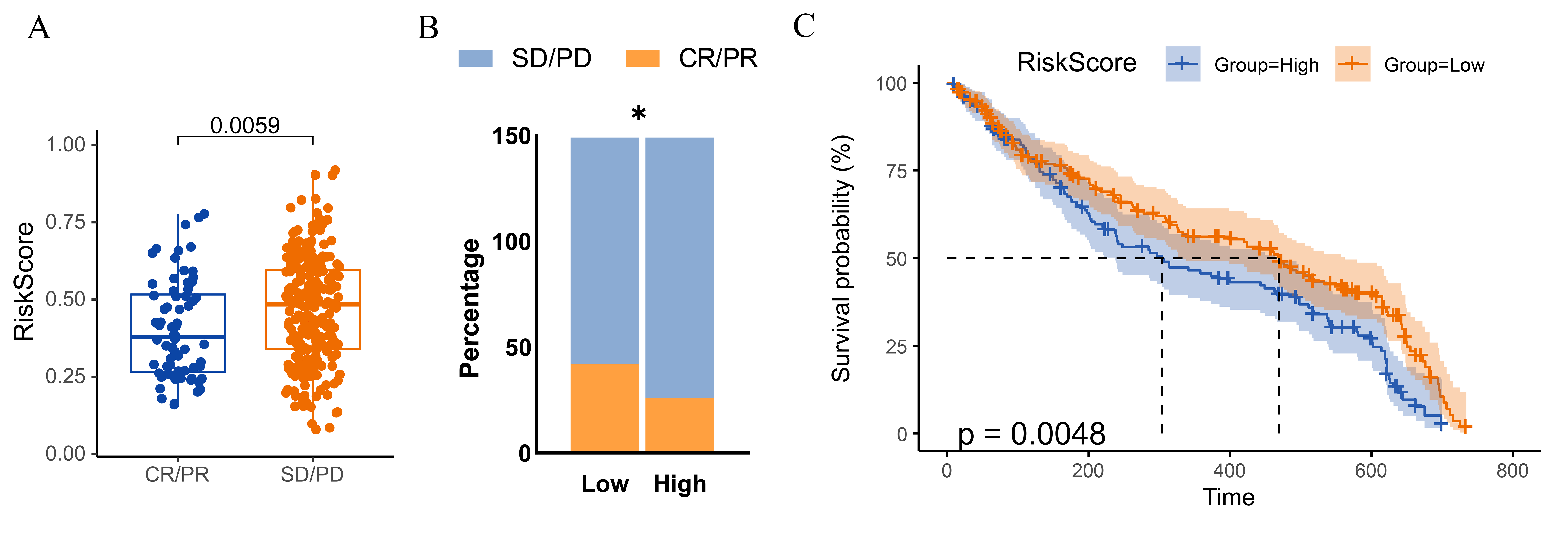

Supplement: Supplementary Figure 8 — Validation of the risk score in the IMvigor210 cohort. (A) Boxplot depicting the risk scores between SD/PD and CR/PR groups in the IMvigor210 cohort. (B) Differences in the number responders between high- and low-risk groups in the IMvigor210 cohort. (C) Kaplan-Meier curves displaying prognostic differences between high- and low-risk groups in the IMvigor210 cohort. *, P < 0.05; **, P < 0.01; ***, P < 0.001; ****, P < 0.0001. [file Image_8.tif]
